# Enhanced Estimation of Axial Compressive Strength for CFRP Based on Microscale Numerical Simulation and the Response Surface Method

**DOI:** 10.3390/ma17020478

**Published:** 2024-01-19

**Authors:** Honoka Yoshida, Huachao Deng, Jun Koyanagi

**Affiliations:** 1 Graduate School of Science, Tokyo University of Science, 6-3-1, Niijuku Katsushika-ku, Tokyo 125-8585, Japan; 8218091@alumni.tus.ac.jp; 2 Department of Materials Science and Technology, Tokyo University of Science, 6-3-1, Niijuku Katsushika-ku, Tokyo 125-8585, Japan; dhc19911202@rs.tus.ac.jp

**Keywords:** CFRP, compressive strength, response surface method

## Abstract

Compressive strength is one of the most important properties of carbon fiber reinforced plastics (CFRP). In this study, a new method for predicting the axial compressive strength of CFRP using the response surface method is developed. We focused on a microbuckling model to predict the compressive strength of unidirectional fiber composites. For the microbuckling model, axial shear properties are required. To obtain the compressive strength for various material properties, we perform individual shear tests and numerical simulations, but these require enormous computational costs and extended time. To address the issue of computational cost, in this study, we propose a new method to predict compressive strength using the response surface method. First, we perform shear simulation in a microscale fracture model for unidirectional CFRP with various parameters of the fiber and resin properties. Based on the results of the shear simulation, the response surface method is used to evaluate and develop prediction equations for the shear properties. This method allows for the study of the objective values of the parameters, without significant computational effort. By comparing both the results predicted from the response surface method (RSM) and the simulation results, we verify the reliability of the prediction equation. As a result, the coefficient of determination was higher than 94%, and the validity of the prediction method for the compressive strength of CFRP using the response surface method (RSM) developed in this study was confirmed. Additionally, we discuss the material properties that affect the compressive strength of composites comprised of fibers and resin. As a result, we rank the parameters as follows: fiber content, elastic modulus after resin yield, yield stress, and initial elastic modulus.

## 1. Introduction

Composite materials are expected to be useful for various industries. Carbon composite materials are now being employed in the aerospace industry [1,2,3,4,5,6,7,8]. Recently, carbon fiber reinforced plastics (CFRP) have been used in automobiles [9,10] because they provide specific energy absorption through the expression of compressive fracturing and delamination [11,12]. To apply CFRP, compressive failure should be carefully considered, as a proper estimation of the compressive strength allows for the efficient design of structures. To explore the longitudinal compression performance of composite materials, several longitudinal compressive failure experiments have been conducted [13] on unidirectional CFRP to investigate the failure process and failure mechanisms.

Over the past three decades, in studies regarding the compressive fracture of unidirectional composites [14,15,16,17,18,19], several types of possible failure modes, such as the Euler buckling or macrobuckling of the specimen, the crushing of the specimen end, longitudinal splitting, interfacial failure, the elastic microbuckling of fibers, the plastic microbuckling of fibers in a kinking mode, and the shear failure of the specimen, have been observed and reported [13]. Among all failure modes, the fiber microbuckling failure mode is recognized as the dominant compressive failure mechanism [20]. Additional studies on CFRP compressive failure can be found in References [21,22,23,24,25,26].

In this study, the compressive strength of CFRP was determined using the fiber microbuckling model proposed by Berbinau et al. [27,28]. The Berbinau fiber microbuckling model is based on the initial fiber waviness, and compressive failure is most likely caused by the local instability of the fibers embedded in the resin. The undulation of the fibers under compressive loading lead to failure.

Berbinau et al. modeled the initial fiber waviness using the sine function $v_{0}\left(x\right)$ as defined below, where $V_{0}$ is the amplitude of the initial fiber waviness and $\lambda_{0}$ is its half wavelength. When a compressive load was applied, the fiber deformed into a sine function $v\left(x\right)$.

(1) $$v_{0}\left(x\right)=V_{0}\sin\left(\frac{\pi x}{\lambda_{0}}\right)$$

(2) $$v\left(x\right)=V\sin\left(\frac{\pi x}{\lambda }\right)$$

Based on the assumption that the fibers buckle in the phase, all fibers deform in the same manner; therefore, if  p=q=0, this can be noted as Equation (3).

(3) $$\frac{d^{2}M}{dx^{2}}+P\frac{d^{2}v}{dx^{2}}+\frac{dm}{dx}=0$$

Considering all forces, deflection curves, moments, shear forces, and deformations applied to the fiber owing to compressive loading, Equation (4) was derived for the microbuckling model. The shear stiffness G is given in Equation (5).

(4) $$\frac{V}{V_{0}}={\left[1-\frac{P}{E_{f}I{\left(\pi /\lambda \right)}^{2}-A_{f}G}\right]}^{-1}$$

(5) $$G_{12}^{ep}\left(\gamma \right)=G_{12}^{e}exp\left(-\frac{G_{12}^{e}\gamma }{\tau_{y}}\right)+G_{12}^{p}exp\left(-\frac{G_{12}^{p}\gamma }{\tau_{ult}-\tau_{y}}\right)$$

Here, I is the moment of inertia of area, $\;A_{f}$ is the fiber cross section, $G_{12}^{ep}$ is the composite shear modulus, $G_{12}^{e}$ and $G_{12}^{p}$ are the elastic and plastic out-of-plane shear modulus, respectively, $\;\tau_{y}$ is the yield shear stress, γ is the shear strain, and $\tau_{ult}$ is the shear failure stress.

$V⁄V_{0}$, as shown in Equation (4), increased slowly with the stress σ and then increased exponentially, ultimately reaching the maximum value. In the function shown in Equation (4), we assume that the fiber buckles at the point of the asymptote, defining the stress reached as the compressive strength. In other words, this compressive strength prediction model predicts compressive strength using fiber buckling. As can be seen from Equation (4), the composite shear mechanical properties are necessary to predict the compressive strength using the micro buckling model.

Considering the compressive failure of microbuckling, the out-of-plane shear properties and initial irregular angles of the fibers are important parameters that affect the compressive strength, as shown in a study by Jumahat et al. [13]. The shear properties vary significantly, depending on the material properties of the composite fibers and resin, and these properties must be estimated in individual shear tests or numerical simulations [29]. However, conducting experiments and simulations of various material properties is inefficient because it requires a large amount of computation. In this paper, we propose the response surface method (RSM) as a multivariate statistical method to reduce these computational requirements. the response surface method (RSM) [30,31,32,33,34] is a mathematical and statistical technique [35] that approximates discrete data to a continuous surface using the lowest amount of measurement data. This enables highly accurate predictions using a small number of simulations [36].

This study proposes a new method for predicting the axial compressive strength of composite materials. We address the issue of reducing computational cost, which has been unresolved in previous studies, and develop a prediction model from the perspective of the response surface method, which is different from the conventional approach. Specifically, we propose an efficient and precise method for predicting axial compressive strength by integrating the microbuckling model and the response surface method (RSM). This method enables the prediction of compressive strength, without requiring the performance of simulations each time, allowing for the comparison of the effects of different material properties on the compressive strength. The parameters of the material properties of the fiber and resin were designed based on the experimental method, and an axial shear simulation was performed using a three-dimensional periodic unit cell (3D PUC) [37,38,39,40] model of CFRP. The results obtained from the simulation were applied to the response surface method (RSM) to create regression equations, and the reliability of the regression equations was verified by comparing them with numerical simulation values. Additionally, based on the developed predictive equations, we discuss which material properties within the composite materials comprised of fiber and resin affect the compressive strength.

The added value of this research is that it will provide efficient and accurate predictions when assuming compressive strength in various fiber and resin materials and when considering the materials that should be selected to achieve the target compressive strength. This method is expected to play a role in the design and material selection process.

**Figure 1:**
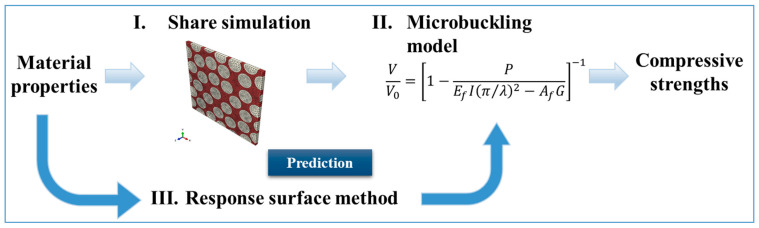
Flow of the calculation of compressive strength in RSM and microbuckling.

Figure 1 shows a brief flow of this study, and I, II, and III are explained. First, shear simulations (I) of CFRP are performed using numerical simulations as a conventional method to calculate compressive strength. The compressive strength can be calculated by applying the obtained shear property results to the equation of the microbuckling model (II). However, using this conventional method, this is not efficient in terms of computational cost and the time required to perform shear simulations for each material, which is an issue. Therefore, this study proposes a novel method of prediction based on the response surface method (III). This method eliminates the need for each simulation, reduces computational costs, and enables the efficient calculation of shear properties. The compressive strength of CFRP can also be obtained by combining the predicted shear properties with the microbuckling model (II).

## 2. Numerical Simulation

**Figure 2:**
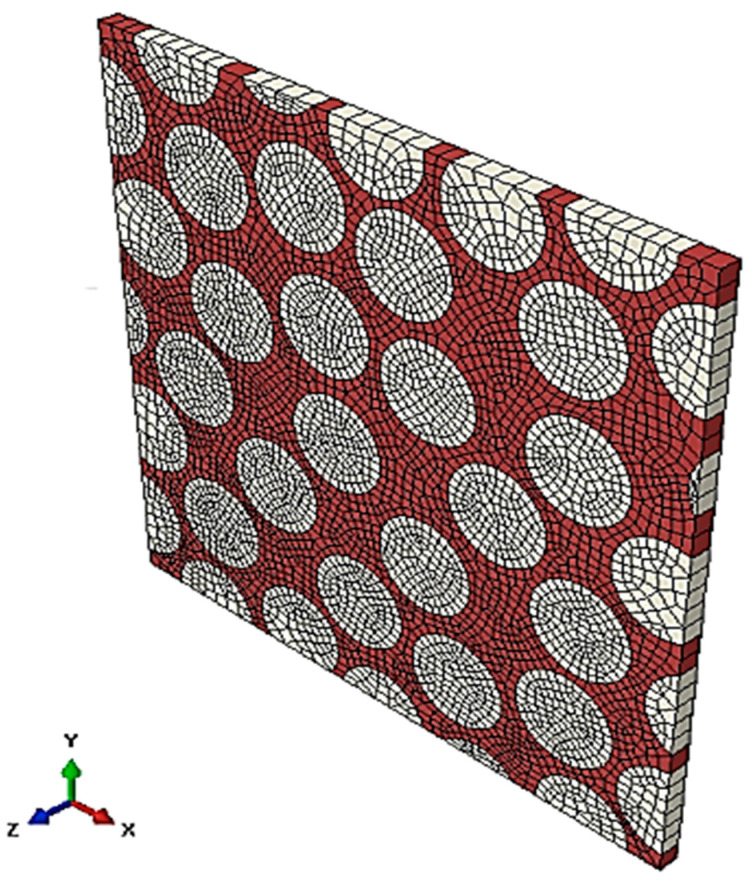
The 3D periodic unit cell (PUC) model of CFRP.

In this section, microscale numerical simulations are described to obtain the shear properties of the composite materials. In this study, the three-dimensional periodic unit cell (3D PUC) model was created using the finite element analysis software, Abaqus 2018/Explicit, as shown in Figure 2.

### 2.1. Numerical Simulation Model

This finite element model comprises a resin, carbon fibers, and an interface between the fibers and the resin. In Figure 2, the area shown in red is the resin, and the area shown in beige is the carbon fiber. The model contained 29 fibers, with a diameter of 6 μm. Both the height and width of the model were approximately 39 μm, and the fiber volume ratio was 54%. Eight-node elements (C3D8) were used to discretize the model in three-dimensional space. The number of elements was 38,322, and the number of nodes was 173,760. For the FEM mesh density in this study, the mesh size dependence is considered to be small because the stress strength field is not pronounced in the damage analysis.

**Figure 3:**
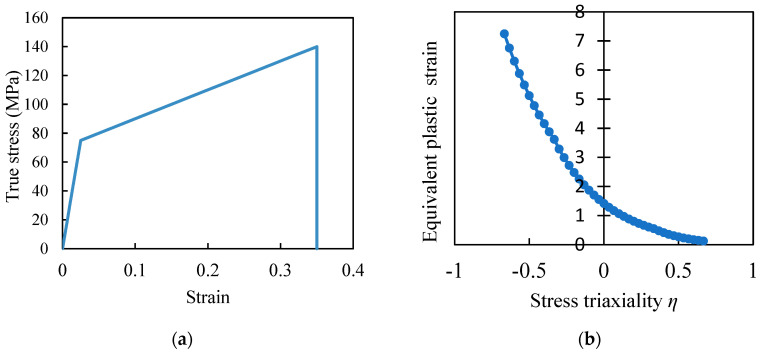
(**a**) Bi-linear assumption of the resin; (**b**) failure criterion for the resin.

The material properties of the 3D PUC are as follows. In this FEM model, the material properties are defined as anisotropic elastic materials for fibers and isotropic elastoplastic materials for resins, considering continuous damage mechanics. These material properties were assumed based on those suggested in Ref. [29], for fiber properties, and in References [20,38,41], for resin properties. The fibers were modeled as anisotropic elastic materials, as listed in Table 1. We defined the Young modulus (E_1_, E_2_, E_3_), Poisson’s ratio (n_12_, n_13_, n_23_), and shear modulus (G_12_, G_13_, G_23_) for the X, Y, and Z directions with respect to the principal axes [40]. The Poisson’s ratio is dimensionless; thus, the units are not listed. Mechanical properties of the resin are described in Table 2 and Figure 3. Resin is an isotropic and elastoplastic material incorporating continuous damage mechanics [41], as shown in Figure 3. Figure 3a shows the stress–strain curve illustrating the deformation behavior of the resin. The plastic damage occurrence criterion was considered for evaluating resin damage. According to this criterion, the equivalent plastic strain at the onset of damage is described as a function of the stress triaxiality and strain rate, as illustrated in Figure 3b.

**Figure 4:**
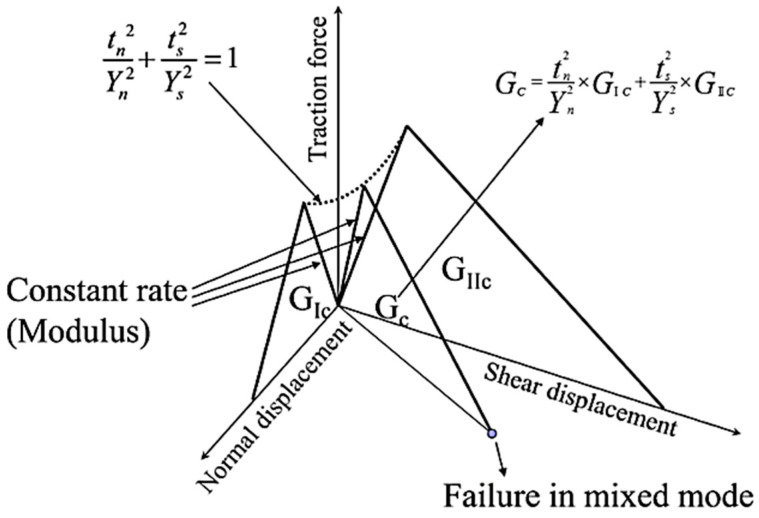
Traction separation law for cohesive behavior under mixed-mode.

Additionally, to consider the interfacial failure between the fiber and the resin, a cohesive element was introduced to model the interfacial behavior and strength [18,19]. The cohesive element is defined using the traction separation law under the mixed mode, as shown in Figure 4.

The traction separation behavior is defined by associating the traction, which acts on the node between the resin and fiber, with the distance between them. In Figure 4, the cohesive element parameters $t_{n}$, $t_{s}$, $Y_{n}$, and $Y_{s}$ are the interfacial tensile stress, the interfacial shear stress, the pure tensile stress, and the pure shear strength, respectively, and $G_{c}$ is their fracture toughness.

The pure shear strength of the cohesive element was assumed to be √2 times larger than the tensile strength [42], and we predicted that the interface shear strength would be 160 MPa. The shear fracture strength of the interface and the mode II fracture toughness ($G_{IIc}$), which is an indicator of material resistance, are presented in Table 3.

### 2.2. Numerical Simulation Results

**Figure 5:**
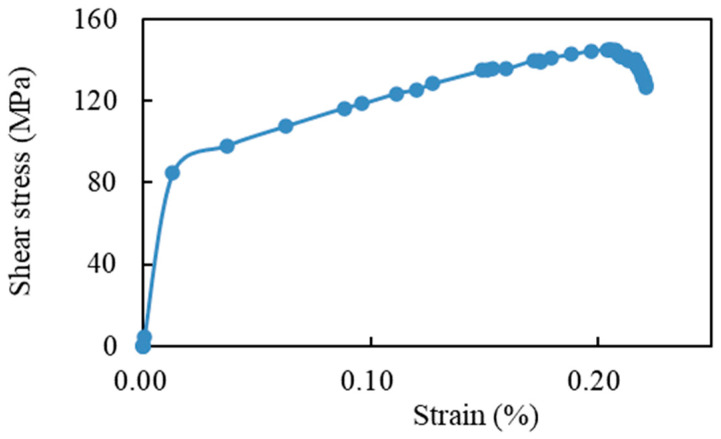
Results of the 3D PUC out-of-plane shear simulation.

This section presents the results of the fiber direction shear simulation using the 3D PUC model. The stress–strain diagrams obtained from the axial shear simulation are shown in Figure 5. The shear simulation results indicate that the material underwent yielding at a certain stress, followed by plastic deformation, which finally led to failure. From the shear stress–strain relationship shown in Figure 5, the yield stress, fracture stress, and the elastic and plastic modulus were derived, and these values are shown in Table 4. The respective values in Table 4 are the elastic and plastic out-of-plane shear modulus; $G_{12}^{e}$ and $G_{12}^{p}$; the yield shear stress, $\tau_{y}$; and the shear failure stress, $\tau_{ult}$.

These four parameters are the mechanical properties necessary to obtain the CFRP unidirectional compressive strength. Subsequently, the four shear properties listed in Table 4 were applied to the microbuckling model to calculate the compressive strength.

### 2.3. Compressive Strength Using the 3D PUC Model and the Microbuckling Model

**Figure 6:**
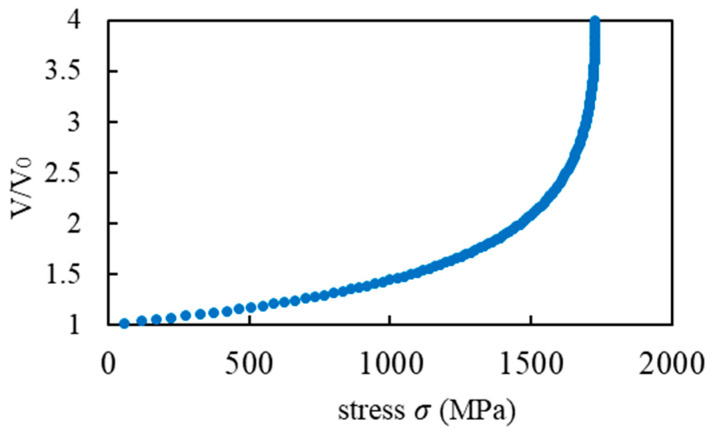
Results for the microbuckling model.

To obtain the compressive strength, four shear property values were applied to the microbuckling model. The microbuckling model is represented by Equations (6)–(8) [13,27,28], and a graph showing the left-hand side, $V⁄V_{0}$, as the vertical axis and the right-hand side as the horizontal axis is shown in Figure 6. The material parameters for the microbuckling formula were calculated based on the analysis conditions described in Section 2.1, and these are shown in Table 5.

(6) $$\frac{V}{V_{0}}={\left[1-\frac{P}{E_{f}I{\left(\pi /\lambda \right)}^{2}-A_{f}G_{12}^{ep}}\right]}^{-1}$$

(7) $$\sigma =\frac{PV_{f}}{A_{f}}$$

(8) $$G_{12}^{ep}\left(\gamma \right)=G_{12}^{e}exp\left(-\frac{G_{12}^{e}\gamma }{\tau_{y}}\right)+G_{12}^{p}exp\left(-\frac{G_{12}^{p}\gamma }{\tau_{ult}-\tau_{y}}\right)$$

In Figure 6, $V/V_{0}$ slowly increased with the increase in the applied stress σ, and then exponentially grew until it reached maximum stress. Equation (6) assumes that V in the function increases rapidly, and that the fiber buckles at the point of the asymptote. The stress at the asymptote point was defined as the compressive strength. In terms of the material properties described in Section 2.1, this graph shows that the predicted compressive strength was 1730 MPa.

In this section, microscale numerical simulations conducted using the 3D-PUC model are described, and their shear properties are applied to a microbuckling model to calculate the compressive strength. This method has been used as a valid compressive strength evaluation method, and the compressive strength of 1730 MPa shown in Figure 6 is roughly consistent with the experimental results of Sawamura et al. [20,29]. However, this method, particularly the shear simulation to calculate shear properties, requires considerable time and computational cost.

To evaluate the compressive strength of various materials, it is necessary to perform a shear simulation for each material, which is highly inefficient. Therefore, we propose a compressive strength evaluation method using the response surface method as a faster and more efficient method for compressive strength evaluation.

## 3. The Response Surface Method (RSM)

### 3.1. Introduction

During material development, it is often difficult to evaluate materials by prototyping and experimentation, and numerical simulations are useful to predict material properties and design materials [36]. However, the actual exploration of material properties requires simulation of the microstructure of the material at the nanoscale, which requires a significant amount of time.

One method to reduce this design time is the response surface method (RSM), which predicts the design space with high accuracy. The response surface method (RSM) is a mathematical and statistical method for representing continuous solutions from discrete data using the minimum amount of measurement data [33]. This method can be used to predict solutions without requiring any simulations.

The RSM is used worldwide in quality engineering, i.e., in product process optimization and variability phenomena [30,31,32,33,34]. Using this method, a significant reduction in the development time can be achieved by replacing conventional experimental and trial-and-error design studies with a combination of simulation and optimization methods. In other words, the RSM is a faster and more efficient method for designing new materials and processes.

In Section 2, we discussed the fact that performing simulations individually requires high computational costs and extensive time periods, and this is very inefficient. To overcome this problem, the RSM has been proposed as a method to reduce computational complexity using multivariate statistical methods. The RSM specifically explores the relationship between response y and factors ($x_{1},x_{2}\ldots ,x_{p}$) by collecting data according to an experimental design.

### 3.2. Central Composite Designs

In this study, using the RSM, we created a prediction equation using the material properties as explanatory variables and the results of shear simulation as objective functions. Based on the prediction equation, the compressive strength was evaluated to determine the material properties that affect it. To apply the RSM, we perform the following steps [36,43]:

1. The variables of main influence are selected, and the boundaries of the experimental domain are set for these variables.
2. Experiments are conducted based on the experimental method design.
3. The coefficients β of the polynomial function are determined through both mathematical and statistical calculations..
4. The goodness of fit of the model is evaluated.
5. The influence of the material is assessed using the desirability function.

In this study, we adopted a polynomial approximated model widely used in the RSM. The coefficients of the function were statistically estimated using the least squares method. The RSM calculations are shown in Equation (9).

(9) $$y_{n}=\beta_{0}+\sum_{i=1}^{k}\beta_{i}x_{i}+\sum_{i=1}^{k}\beta_{ii}x_{i}^{2}+\sum_{i<j}\beta_{ij}x_{i}x_{j}$$

The $y_{n}$ term shows four shear properties for n=1~4, respectively: the elastic and plastic out-of-plane shear modulus; $G_{12}^{e}$ and $G_{12}^{p}$; yield shear stress, $\tau_{y}$; and shear failure, $\tau_{ult}$. $\beta_{0}$ is the constant term, $\beta_{i}$ represents the coefficients of the liner parameters, and $\beta_{ij}$ represents the coefficient of the quadratic parameters. The x term refers to the explanatory variables, which are shown in Table 6, with k=6.

The experimental design is based on these explanatory variables. Although the central composite design, the Behenken design, and three-level factorial design [36] are used as experimental designs for the RSM, the central composite design was adopted in this study. The central composite design adds a center point and an axis point to the design, and all factors are evaluated at five factorial levels (−α. −1, 0, 1, α) to create a model with curved surface properties [36]. The first step in implementing this plan was to standardize the explanatory variables. The standardization is based on Equation (10).

(10) $$x_{i}=\frac{x-\frac{x_{u}+x_{l}}{2}}{\frac{x_{u}-x_{l}}{2}}$$

In Equation (10), $x_{u}$ is the upper level, $x_{l}$ is the lower level, and $x_{i}$ is the level value after standardization. The $x_{u}$ and $x_{l}$ values need to be set for each parameter, and realistic values for each material property are set based on previous experiments and research [38,39,40]. The explanatory variables at the five-factor level${\;x}_{i}$ (−α. −1, 0, 1, α) are shown in Table 7. In this study, the value of α was set to equalize the range of variation of the factors. In addition, the points of the experimental data were strategically placed on a sphere of radius √2, where the value of α was set to √2.

These explanatory variables were randomized to construct an experimental matrix design, resulting in 77 experimental designs. Based on this central composite design, simulations were performed to calculate the objective variables and shear properties. According the calculated shear properties and material property parameters (77 in total), a regression model was constructed to explain the response variables. Specifically, the regression coefficient β of Equation (9) was calculated using the statistical software JUSE-StatWorks/V5 (Institute of the Japanese Union of Scientists and Engineers).

### 3.3. Determination of β

A common technique for calculating the coefficient β is using the method of least squares [36]. The coefficient β of the model is adjusted with the aim of minimizing the residuals between the predicted and measured values from a hypothetical regression model. When the number of experiments is n and the number of variables is  k, the predictive model is represented by the matrix in Equations (11) and (12).

(11) y=Xβ+ε

(12) $$\left[\begin{array}{c} y_{1}\\ y_{2}\\ \vdots \\ y_{n} \end{array}\right]=\left[\begin{array}{ccccc} y_{1} & x_{11} & x_{12} & \cdots & x_{1k}\\ y_{2} & x_{21} & x_{\mathrm{22}} & \cdots & x_{2k}\\ \vdots & \vdots & \vdots & \ddots & \vdots \\ y_{n} & x_{n1} & x_{n2} & \cdots & x_{nk} \end{array}\right]\left[\begin{array}{c} \beta_{0}\\ \beta_{1}\;\\ \vdots \\ \beta_{k} \end{array}\right]+\left[\begin{array}{c} \varepsilon_{1}\\ \varepsilon_{2}\\ \vdots \\ \varepsilon_{n} \end{array}\right]\;$$

By minimizing the sum of error squares, the unbiased estimator b of the coefficients β is obtained as follows:

(13) $$b={\left(X^{T}X\right)}^{-1}X^{T}y$$

The appropriateness of the regression model was determined using the coefficient of determination defined by Equation (14). The coefficient of determination ranges from 0 to 1, and is less than 1 if there are residuals.

(14) $$R^{2}=\frac{SSR}{S_{yy}}=1-\frac{SSE}{S_{yy}}$$

Here, *SSE* and *SSR* denote the residual and regression sums of squares, respectively. In this study, the coefficient β is calculated using differentiation and optimization algorithms in the statistical software JUSE-Stat Works/V5, leading to the response surface equation. Table 8 shows the β values calculated using Equations (12) and (13). In Equation (9), $\beta_{0}$ represents the constant term, $\beta_{i}$ the coefficient of the linear term, $\beta_{ii}\;$the coefficient of the quadratic term, and $\beta_{ij}$ the coefficient of the interaction term. The values of i and j range from 1 to 6, corresponding to the explanatory variables listed in Table 6.

The coefficients β of these linear, quadratic, and interaction terms indicate how much each parameter contributes to the shear properties. The coefficients of the linear terms exhibited a strong linear influence on the parameters and shear properties, whereas the coefficients of the quadratic terms exhibited a curvilinear relationship. The coefficients of the interaction terms emphasize the combined actions of the parameters. In Table 8, a comparison of the coefficients shows that each shear property is influenced by different material property parameters, which tend to vary with the shear properties.

## 4. Prediction of Compressive Strength Using RSM

### 4.1. Prediction Equation of Compressive Strength

**Figure 7:**
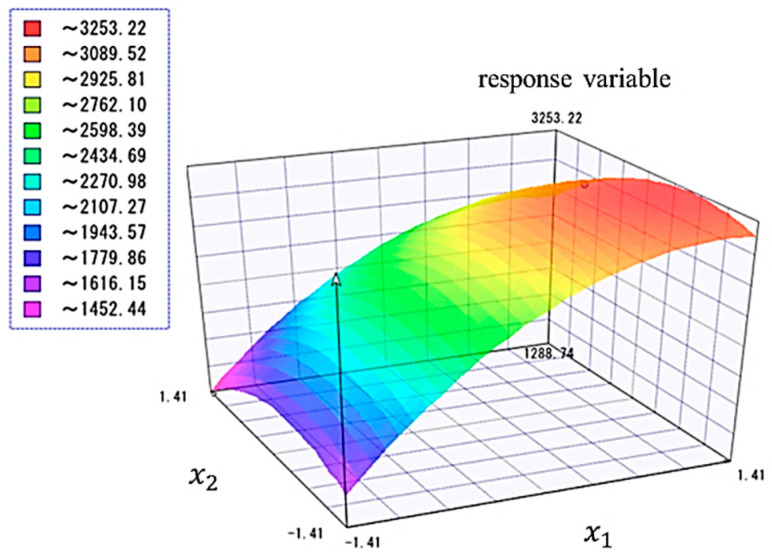
Graph of RSM prediction formula for $x_{1}$ and $x_{2}$.

In this section, regression equations are developed based on the estimated coefficients β and Equation (9) for the four shear properties ($G_{12}^{e}$, $G_{12}^{p}$, $\tau_{y}$, and $\tau_{ult}$). The RSM prediction equation can be graphed by plotting any two parameters on the x and y axis and the response variable on the z axis. Figure 7 shows an example of a response surface diagram. The x axis of this diagram shows the $x_{1}$ fiber content, and the y axis shows $x_{2}$ initial elastic modulus.

This graph allows for a quantitative and visual understanding of how the parameters and their interactions affect the response variable. Owing to these advantages, the RSM is widely used in various fields such as experimentation, process optimization, and product improvement [30,34,36].

### 4.2. The Validity of the RSM Equation

This section presents a validation of the reliability of the prediction equations derived using the RSM. Specifically, the values obtained from the prediction equation using the RSM were compared with those obtained from the numerical simulation described in Section 2. The x axis represents the shear property values obtained from the prediction equation, and the y axis represents the values obtained from the numerical simulation.

In addition, to assess the accuracy of the forecasting equation, we focused on whether there was a large amount of data close to y=x. The stronger this tendency, the more reliable the prediction equation. In this study, we adopted the coefficient of determination as an indicator to verify the accuracy of the forecasting equation. The coefficient of determination is an important indicator of the predictive ability of regression models. Specifically, it indicates the proportion of variance in the data explained by the regression model; the closer the value is to 1, the better the model’s predictions fit the actual data.

**Figure 8:**
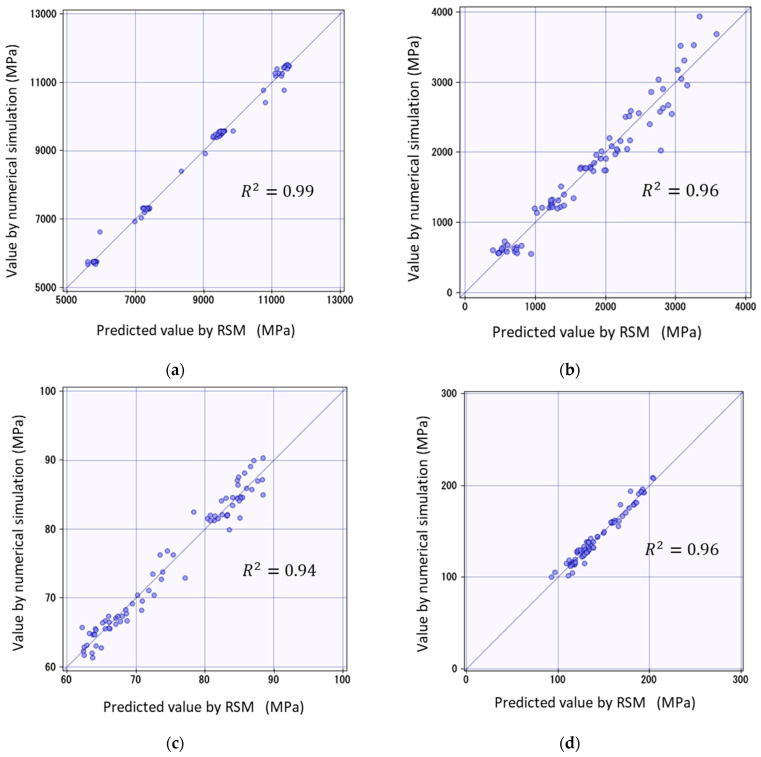
Comparison of RSM and numerical simulation: (**a**) elastic shear modulus $G_{12}^{e}$; (**b**) plastic shear modulus $G_{12}^{p};\;$(**c**) out-of-plane share yield stress $\tau_{y}$; (**d**) out-of-plane share stress $\tau_{ult}$.

The coefficients of determination $R^{2}$ for the four graphs are listed in Figure 8. In all prediction equations, the coefficient of determination exceeded 94%. Specifically, the average coefficient of determination for each prediction equation was 96.3%, with the highest reaching 99%. Thus, the prediction model agreed well with the actual data, suggesting that a highly accurate response surface equation was obtained.

**Figure 9:**
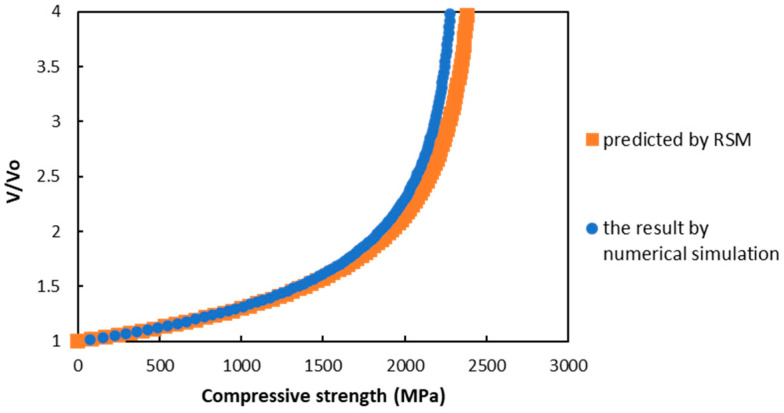
Comparison of RSM and numerical simulation for compressive stress.

Additionally, we confirmed the validity of the compressive strength prediction using the prediction equation from the RSM by comparing the predicted and measured values. We applied the predicted shear values to the microbuckling model and calculated the compressive strength. The microbuckling equations are based on Equation (15), as described in Section 1. Figure 9 shows an example of the results of comparing the calculated compressive strength predicted value and the actual measured value. This is the result of the prediction of material properties at factor level $x_{i}=0$ in Table 7, i.e., $x_{1}=60$; $x_{2}=3500$; $x_{3}=65$; $x_{4}=500$; $x_{5}=120$; $x_{6}=140$.

From Figure 9, it can be confirmed that for any given material, the predicted and measured values are in close agreement. Additionally, the error is significantly small (within 10%), confirming that the prediction equation based on the RSM developed in this study is highly reliable.

(15) $$\frac{V}{V_{0}}={\left[1-\frac{P}{E_{f}I{\left(\pi /\lambda \right)}^{2}-A_{f}G}\right]}^{-1}\;G_{12}^{ep}\left(\gamma \right)=G_{12}^{e}exp\left(-\frac{G_{12}^{e}\gamma }{\tau_{y}}\right)+G_{12}^{p}exp\left(-\frac{G_{12}^{p}\gamma }{\tau_{ult}-\tau_{y}}\right)$$

### 4.3. The Factors Affecting Compressive Strength

In this section, we discuss the influence of the parameters on the compressive strength prediction using the RSM and microbuckling model. In the discussion, we evaluate the relationship by optimizing the desirability function. The scale is such that 0 indicates a totally undesired reaction, and the closer it is to 1, the more desirable the reaction [36,43]. For each response variable, the upper and lower limits, $U_{t}$ and $L_{t}$, are determined, as is the coefficient k which determines the shape of the desirability function [44], as shown in Equation (16).

(16) $$bd_{t}\left(x_{1},\ldots ,x_{p}\right)=\left\{\begin{array}{c} 0\;:if\;y<1\;\\ {\left(\frac{{\hat{\mu }}_{t}\left(x_{1},\ldots ,x_{p}\right)-L_{t}}{U_{t}-L_{t}}\right)}^{k}\;:if\;L\leq y\leq T\\ 1\;:if\;y>T\; \end{array}\right.$$

**Figure 10:**
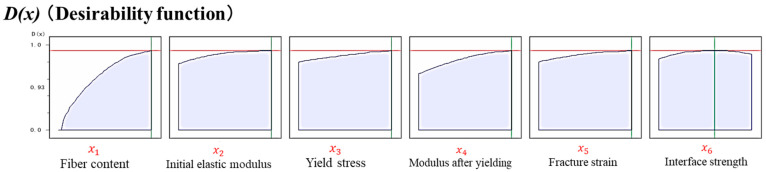
Desirability function for each material property.

We evaluated the effect of each material property on the compressive strength. The desirability functions were calculated from the compressive strength data derived from the RSM and microbuckling models, and are shown in Figure 10. This figure shows the factors $x_{1}\sim x_{6}$ on the horizontal axis, the predicted compressive strength values on the vertical axis, and the desirability function $D(x_{1},x_{2},x_{3},x_{4},x_{5},x_{6})$.

The desirability functions D(x) for each material property are compared in Figure 10. The parameters whose desirability functions change significantly are the most influential, in the following order: fiber content $\;x_{1}$, modulus after yielding $\;x_{4}$, yield stress $x_{3}$, and initial elastic modulus $x_{2}$.

**Figure 11:**
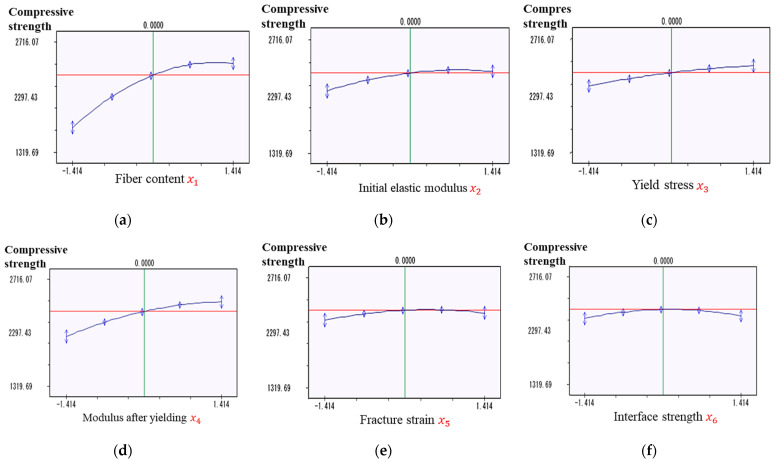
Compressive strength for each material property: (**a**) fiber content; (**b**) initial elastic modulus; (**c**) yield stress; (**d**) modulus after yielding; (**e**) fracture strength; (**f**) interface strength.

Subsequently, we focused on each material property$\;x_{1}\;to\;x_{6}$ individually. The effects of the six explanatory variables on the compressive strength are shown in Figure 11. The limits for the material property parameters considered in this study were established based on the results of previous studies [38,39,40], as described in Section 3. The horizontal axis shows the normalized values of the parameters, and the vertical axis shows the compressive strength.

First, we focused on the fiber content, as shown in Figure 11a. The graph shows that the compressive strength surges as the fiber content increases from 46% to 70%. The rate of change tended to increase rapidly at first, and then moderately. From 70% to 74%, a slight decrease was observed. Therefore, a fiber content in the range of 60–70% is optimal, and the effect may decrease if the fiber content exceeds this range.

We then examined the initial modulus of elasticity $x_{2}$, as shown in Figure 11b. As the initial modulus of elasticity increased from 2800 to 4200 MPa, the compressive strength also tended to increase. Additionally, the rate of change was relatively high at the beginning, and then became moderate.

We show the yield stress $\;x_{3}$ in Figure 11c. The graph shows that as the yield stress increased from 51 to 79 MPa, the compressive strength also increased gradually. The rate of change was relatively high at the beginning, and then converged. Overall, the yield stress affected the compressive strength; however, the extent of this effect was not as significant as that of the other parameters.

The modulus after yielding $x_{4}$ is shown in Figure 11d. The graph shows a trend of increasing compressive strength with increasing modulus after yielding. In the range of $x_{4}$ considered in this study, the compressive strength varied widely from approximately 1950 MPa to 2410 MPa. This indicates that varying the modulus after yielding may significantly affect the compressive strength.

Finally, we show the fracture strength $x_{5}$ and interface strength $x_{6}$ in Figure 11e and Figure 11f, respectively. The fracture and interfacial strengths had some influence on the compressive strength; however, the range of variation was narrower than that of the other parameters. Therefore, the influence of these properties on the compressive strength is limited, and other factors are thought to have a significant effect on the compressive strength. As a result, the effects of the fracture strength and interfacial strength are considered relatively negligible.

### 4.4. Prioritizing Factors Affecting Compressive Strength

Based on this discussion, we ranked the parameters that were most likely to affect the compressive strength. The order is: fiber content $\;x_{1}$, modulus after yielding of resin $\;x_{4}$, yield stress of resin $\;x_{3}$, and initial elastic modulus of resin $x_{2}$.

These rankings are based on the rate of change in the compressive strength and desirable function described in Section 4.3. For the range of parameters considered in this study, the fiber content showed the highest change in the compressive strength, followed by the modulus after yielding. The yield stress and initial elastic modulus of the resin are also considered to be factors that affect the compressive strength. This indicates that the resin properties of the composites were dominant during compression.

However, modifying these parameters is difficult in practice and highly dependent on the materials used, as well as the manufacturing process. For example, the resin properties may be modified by material selection and heat treatment; however, the manufacturing process, design implications, and costs should be considered.

## 5. Conclusions

In this study, a new prediction method for the axial compressive strength of composite materials was developed using the RSM method and a microbuckling model. The parameters of the material properties of the fiber and resin were calculated based on the design of the experimental method. An axial shear simulation was performed using the 3D PUC model of CFRP, and the prediction equation was formulated by applying the results obtained from the analysis to the RSM. The reliability of the prediction equation was compared with the numerical simulation results, and the validity of the equation was evaluated by determining the goodness of fit of the prediction model using the coefficient of determination. In all prediction equations, the coefficient of determination exceeded 94%, indicating a high goodness of fit. Specifically, the average coefficient of determination for each prediction equation was 96.3%, with the highest reaching 99%. These figures demonstrate the exceptional performance of the proposed method as a predictive model, fitting exceptionally well with the actual data. Therefore, the validity of the prediction method for the compressive strength of CFRP using the RSM method developed in this study was confirmed. Additionally, we discuss the material properties that affect the compressive strength of composites comprised of fibers and resin. Consequently, we ranked the parameters most likely to influence the compressive strength as follows: fiber content, elastic modulus after resin yield, yield stress of resin, and the initial elastic modulus of resin.

## Figures and Tables

**Table 1:** Mechanical properties of the fiber.

E_1_	E_2_	E_3_	n_12_	n_13_	n_23_	G_12_	G_13_	G_23_
14 GPa	14 GPa	294 GPa	0.35	0.02	0.02	5 GPa	18 GPa	18 GPa

**Table 2:** Mechanical properties of the resin.

Resin modulus	3.6 GPa
Resin Poisson’s ratio	0.34

**Table 3:** Cohesive properties of the 3D PUC model.

$Y_{S}=\sqrt{2}Y_{n}$	160 MPa
$G_{\mathrm{IIC}}$ *=2G*_IC_ $=G_{C}$	0.008 N/mm

**Table 4:** The out-of-plane shear properties obtained from the 3D PUC simulation.

$G_{12}^{e}$	Elastic shear modulus	6530 MPa
$G_{12}^{p}$	Plastic shear modulus	300 MPa
$\tau_{y}$	Out-of-plane shear yield stress	85 MPa
$\tau_{ult}$	Out-of-plane shear strength	140 MPa

**Table 5:** Material parameters in the microbuckling method.

Material Properties		
Fiber diameter	$d_{f}$	6 μm
Fiber content	$V_{f}$	54%
Fiber cross section	$A_{f}$	28.3 μm^2^
Fiber Young modulus	$E_{f}$	294 GPa
Fiber half-waviness	$\lambda =10d_{f}$	60 μm
Moment of inertia of area	$I=d_{f}^{4}\pi /64$	63.6 μm^4^

**Table 6:** The explanatory variables.

$x_{i}$	Explanatory variables
$x_{1}$	Fiber content
$x_{2}$	Initial elastic modulus
$x_{3}$	Yield stress
$x_{4}$	Modulus after yielding
$x_{5}$	Fracture strength
$x_{6}$	Interface strength

**Table 7:** Explanatory variables divided into five levels.

	Explanatory Variables	−α	−1	0	+1	+α
$x_{1}$	Fiber content	45	50	60	70	75
$x_{2}$	Initial elastic modulus	2800	3000	3500	4000	4200
$x_{3}$	Yield stress	51	55	65	75	79
$x_{4}$	Modulus after yielding	75	200	500	800	925
$x_{5}$	Fracture strength	92	100	120	140	148
$x_{6}$	Interface strength	55	80	140	200	225

**Table 8:** Calculated value of β in Equation (9).

		Elastic Shear Modulus	Plastic in Plane Shear Modulus	Yield Share Stress	Failure Shear Stress
$\beta_{i}$	β _0_	9871	2310	73.89	162
	β _1_	1910	436	0.44	−3
	β _2_	850	−51	0.67	1.03
	β _3_	60	79	9.27	−0.65
	β _4_	35	752	−0.45	−2.98
	β _5_	31	−39	1.25	17.69
	β _6_	35	−62	0.95	15.46
$\beta_{\mathrm{ii}}$	β _1_^2^	−606	−226	−0.39	−3.65
	β _2_^2^	−159	−145	−0.13	−0.59
	β _3_^2^	−166	−31	0.72	2.67
	β _4_^2^	−163	−153	1.91	−3.60
	β _5_^2^	−163	−40	−0.12	−3.69
	β _6_^2^	−171	−29	−0.85	−13.01
$\beta_{\mathrm{ij}}$	β _1_ β _2_	95	−27	0.17	0.25
	β _1_ β _3_	31	−3	−0.57	−0.56
	β _1_ β _4_	13	105	0.44	−0.86
	β _1_ β _5_	14	−2	0.18	−0.06
	β _1_ β _6_	39	−51	0.25	−3.07
	β _2_ β _3_	25	−25	−0.04	0.13
	β _2_ β _4_	17	−24	0.05	0.62
	β _2_ β _5_	13	5	−0.03	0.03
	β _2_ β _6_	9	−15	−0.04	0.01
	β _3_ β _4_	−30	32	−0.24	−3.90
	β _3_ β _5_	−24	−13	0.35	3.60
	β _3_ β _6_	−30	−16	−0.51	−2.31
	β _4_ β _5_	−29	−59	0.17	2.12
	β _4_ β _6_	−27	−42	−0.79	−6.04
	β _5_ β _6_	−33	91	0.23	13.45

## Data Availability

Data are contained within the article.
